# Anticancer Activities of Pterostilbene-Isothiocyanate Conjugate in Breast Cancer Cells: Involvement of PPARγ

**DOI:** 10.1371/journal.pone.0104592

**Published:** 2014-08-13

**Authors:** Kumar Nikhil, Shruti Sharan, Abhimanyu K. Singh, Ajanta Chakraborty, Partha Roy

**Affiliations:** 1 Molecular Endocrinology Laboratory, Department of Biotechnology, Indian Institute of Technology Roorkee, Roorkee, Uttarakhand, India; 2 Department of Macromolecular Structures, Centro Nacional de Biotecnologia (CNB-CSIC), Campus de Cantoblanco, Madrid, Spain; University of Pittsburgh, United States of America

## Abstract

Trans-3,5-dimethoxy-4′-hydroxystilbene (PTER), a natural dimethylated analog of resveratrol, preferentially induces certain cancer cells to undergo apoptosis and could thus have a role in cancer chemoprevention. Peroxisome proliferator-activated receptor γ (PPARγ), a member of the nuclear receptor superfamily, is a ligand-dependent transcription factor whose activation results in growth arrest and/or apoptosis in a variety of cancer cells. Here we investigated the potential of PTER-isothiocyanate (ITC) conjugate, a novel class of hybrid compound (PTER-ITC) synthesized by appending an ITC moiety to the PTER backbone, to induce apoptotic cell death in hormone-dependent (MCF-7) and -independent (MDA-MB-231) breast cancer cell lines and to elucidate PPARγ involvement in PTER-ITC action. Our results showed that when pre-treated with PPARγ antagonists or PPARγ siRNA, both breast cancer cell lines suppressed PTER-ITC-induced apoptosis, as determined by annexin V/propidium iodide staining and cleaved caspase-9 expression. Furthermore, PTER-ITC significantly increased PPARγ mRNA and protein levels in a dose-dependent manner and modulated expression of PPARγ-related genes in both breast cancer cell lines. This increase in PPARγ activity was prevented by a PPARγ-specific inhibitor, in support of our hypothesis that PTER-ITC can act as a PPARγ activator. PTER-ITC-mediated upregulation of PPARγ was counteracted by co-incubation with p38 MAPK or JNK inhibitors, suggesting involvement of these pathways in PTER-ITC action. Molecular docking analysis further suggested that PTER-ITC interacted with 5 polar and 8 non-polar residues within the PPARγ ligand-binding pocket, which are reported to be critical for its activity. Collectively, our observations suggest potential applications for PTER-ITC in breast cancer prevention and treatment through modulation of the PPARγ activation pathway.

## Introduction

The incidence of cancer, in particular breast cancer, continues to be the focus of worldwide attention. Breast cancer is the most frequently occurring cancer and the leading cause of cancer deaths among women, with an estimated 1,383,500 new cases and 458,400 deaths annually [1]. Many treatment options, including surgery, radiation therapy, hormone therapy, chemotherapy, and targeted therapy, are associated with serious side effects [2]–[5]. Since cancer cells exhibit deregulation of many cell signaling pathways, treatments using agents that target only one specific pathway usually fail in cancer therapy. Several targets can be modulated simultaneously by a combination of drugs with different modes of action, or using a single drug that modulates several targets of this multifactorial disease [6].

Peroxisome proliferator-activated receptors (PPAR) are ligand-binding transcription factors of the nuclear receptor superfamily, which includes receptors for steroids, thyroids and retinoids [7], [8]. Three types of PPAR have been identified (α, β, γ), each encoded by distinct genes and expressed differently in many parts of the body [8]. They form heterodimers with the retinoid X receptor, and these complexes subsequently bind to a specific DNA sequence, the peroxisome proliferating response element (PPRE) that is located in the promoter region of PPARγ target genes and modulates their transcription [9]. PPARγ is expressed strongly in adipose tissue and is a master regulator of adipocyte differentiation [10]. In addition to its role in adipogenesis, PPARγ is an important transcriptional regulator of glucose and lipid metabolism, and is implicated in the regulation of insulin sensitivity, atherosclerosis, and inflammation [10], [11]. PPARγ is also expressed in tissues such as breast, colon, lung, ovary, prostate and thyroid, where it regulates cell proliferation, differentiation, and apoptosis [12]–[14].

Although it remains unclear whether PPAR are oncogenes or tumor suppressors, research has focused on this receptor because of its involvement in various metabolic disorders associated with cancer risk [15]–[17]. The anti-proliferative effect of PPARγ is reported in various cancer cell lines including breast [18]–[21], colon [22], prostate [23] and non-small cell lung cancer [24]. Ligand-induced PPARγ activation can induce apoptosis in breast [13], [20], [25], [26], prostate [23] and non-small cell lung cancer [24], and PPARγ ligand activation is reported to inhibit breast cancer cell invasion and metastasis [27], [28]. Results of many studies and clinical trials have raised questions regarding the role of PPARγ in anticancer therapies, since its ligands involve both PPARγ-dependent and -independent pathways for their action [29].

Previous studies showed that thiazolidinediones can inhibit proliferation and induce differentiation-like changes in breast cancer cell lines both *in vitro* and in xenografted nude mice [13], [30]. Alternately, Abe et al. showed that troglitazone, a PPARγ ligand, can inhibit KU812 leukemia cell growth independently of PPARγ involvement [31]. In addition to *in vitro* studies, *in vivo* administration of PPARγ ligands also produced varying results. The use of troglitazone was reported to inhibit MCF-7 tumor growth in triple-negative immunodeficient mice [13] and in DMBA-induced mammary tumorigenesis [32], and administration of a PPARγ ligand (GW7845) also inhibited development of carcinogen-induced breast cancer in rats [33]. In contrast, a study by Lefebvre et al. showed that PPARγ ligands, including troglitazone and BRL-49653, promoted colon tumor development in C57BL/6JAPCMin/+ mice, raising the possibility that PPARγ acts as a collaborative oncogene in certain circumstances [34]. It thus appears that PPARγ activation or inhibition can have distinct roles in tumorigenesis, depending on the cancer model examined. Hence determining possible crosstalk between PPARγ and its ligand in cancer is critical for the development of more effective therapy.

**Figure 1:**
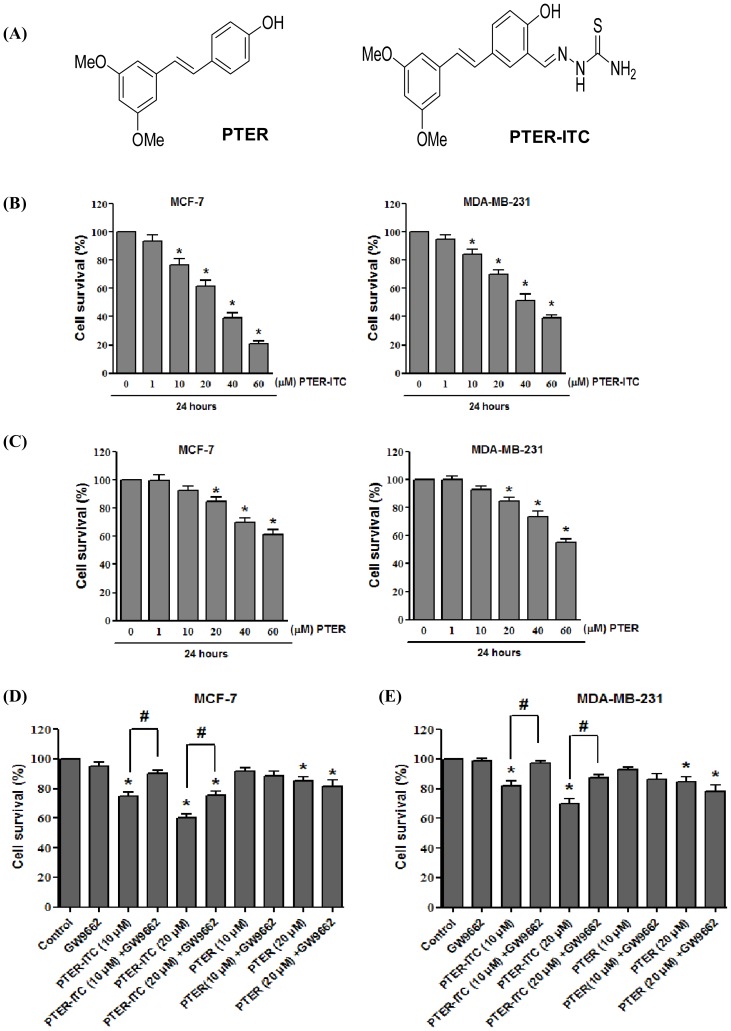
Chemical structure and cytotoxicity assay. (A) Structure of PTER and PTER-ITC conjugate. (B) Cytotoxicity induced by increasing doses of PTER-ITC and (C) PTER in breast cancer cells as determined by MTT assay. (D) Effect of GW9662 on survival of MCF-7 and (E) MDA-MB-231 cells alone and in the presence of PTER-ITC and PTER. Data are shown as mean ± SEM of three independent experiments. * and # indicate statistically significant differences with respect to controls (vehicle-treated) and only PTER-ITC treated groups, respectively. *p*<0.05.

Trans-3,5-dimethoxy-4-hydroxystilbene (PTER) is an antioxidant found primarily in blueberries. This naturally occurring dimethyl ether analog of resveratrol has higher oral bioavailability and enhanced potency than resveratrol [35]. Based on its anti-neoplastic properties in several common malignancies, studies suggest that PTER has the hallmark characteristics of an effective anticancer agent [36]–[40]. Recent research from our laboratory showed that PTER-ITC conjugate (Fig. 1A), a novel class of hybrid compound synthesized by appending an isothiocyanate moiety to the PTER backbone, can induce greater cytotoxicity in tumor cells than PTER alone [41], [42]. In human breast and prostate carcinoma cells, PTER-ITC induces strong anticancer activity at a much lower dose than the PTER parent compound [41], [42].

Here we analyzed the anti-cancer activity of PTER-ITC in MCF-7 and MDA-MB-231 breast cancer cells. As PPARγ mediates anti-tumor activity in a variety of cancer types, we hypothesized that PTER-ITC could modulate the activity of PPARγ pathway in breast cancer cells and inhibit tumor cell growth. Our results show that PTER-ITC induced apoptosis in breast cancer cells through caspase activation, which increased the Bax/Bcl-2 ratio and downregulated survivin. Our molecular docking study also demonstrated that PTER-ITC make contact with amino acids within the ligand-binding pocket of PPARγ that are crucial for its activation. We found that PPARγ activation has an important role in PTER-ITC-induced apoptosis and reduced survivin levels. Our studies thus provide evidence for the usefulness of PTER-ITC in breast cancer therapy involving various pathways, including PPARγ.

## Materials and Methods

### Reagents

All cell culture reagents were from Gibco (Life Technologies, Grand Island, NY). Penicillin, streptomycin, 3-(4,5-dimethyl-2-thiazolyl)2,5diphenyl-2H-tetrazoliumbromide (MTT), cell culture grade dimethyl sulphoxide (DMSO), agarose and all analytical grade chemicals were from Himedia (Mumbai, India). Reverse transcription-polymerase chain reaction (RT-PCR) kits were from Genei (Bangalore, India). 4',6-diamidino-2-phenylindole (DAPI), rosiglitazone, GSK0660 (PPARβ/δ inhibitor), GW9662 (PPARγ inhibitor), PD98059 (MAPK/ERK inhibitor), SB203580 (p38 MAPK kinase inhibitor), SP60025 (JNK inhibitor), Z-VAD-FMK (pan-caspase inhibitor), Z-LEHD-FMK (caspase-9-specific inhibitor), Z-IETD-FMK (caspase-8-specific inhibitor) and BCA protein estimation kits were from Sigma-Aldrich (St. Louis MO). Polyfect transfection reagent was purchased from Qiagen (Valencia CA). Antibodies to caspase-9, Bax, Bcl-2, survivin, PTEN, PPARγ, β-actin, and small interfering RNA (siRNA) against PPARγ (sc-29455) and control (sc-37007; negative control for targeted siRNA transfection experiments; each consists of a scrambled sequence that will not specifically degrade any known cell mRNA) were purchased from Santa Cruz Biotechnology (Santa Cruz, CA). PTER and PTER-ITC conjugate were synthesized in the asymmetric synthesis laboratory (Department of Chemistry, Indian Institute of Technology Roorkee, India) as reported [41].

### Cell lines and culture

Three breast cancer cell lines (MCF-7, MDA-MB-231 and T47D) with distinct characteristics were obtained from the National Center for Cell Science (NCCS; Pune, India). MCF-7 and T47D are estrogen receptor (ER)-positive and lack HER-2 expression, while MDA-MB-231 is ER-negative and has low HER-2 expression. MCF-7 cells express wild-type p53, whereas MDA-MB-231 and T47D express mutant p53. All three cell lines express PPARγ protein. T47D cells were maintained in RPMI medium supplemented with 2 mM L-glutamine, 4.5 g/L glucose and 0.2 U/ml insulin. MCF-7 and MDA-MB-231cells were maintained in Dulbecco's modified Eagle's medium (DMEM) supplemented with 10% fetal bovine serum (heat-inactivated) (both from Invitrogen, Life Technologies) and 1% antibiotic mix (100 U/ml penicillin, 100 µg/ml streptomycin) at 37°C, 5% CO_2_ in a humidified atmosphere.

### Cytotoxicity assays

The anti-proliferative effect of PTER and PTER-ITC was determined by the MTT assay as described [41]. Briefly, MCF-7 and MDA-MB-231 cells were seeded at a density of ∼5×103 cells/well in a 96-well microtiter plate and incubated overnight. Cells were then exposed to increasing PTER and PTER-ITC concentrations (1, 10, 20, 40 and 60 µM) for 24 h. Control cells were treated with 0.1% DMSO (vehicle control). The effect of the inhibitor GW9662 on PTER-ITC-induced cell death was also studied to evaluate involvement of PPARγ activation in this process. After 24 h, cultures were assayed by addition of 20 µl MTT (5 mg/ml) and incubation (4 h, 37°C). MTT-containing medium was then aspirated and 200 µl DMSO was added to dissolve the formazone crystal. Optical density (OD) was measured at 570 nm in an ELISA plate reader (Fluostar Optima, BMG Labtech, Germany). Absorbance values were expressed as percentage of control.

### Change in nuclear morphology of apoptotic cells

Changes in nuclear morphology of apoptotic cells were examined by fluorescence microscopy of DAPI-stained cells. In brief, 0.5×106 cells were seeded in a 6-well plate and incubated (24 h) with 10 and 20 µM concentrations of PTER-ITC in presence and absence of GW9662. For this the cells were pretreated with 10 µM GW9662 for 1 h, followed by treatment with 10 and 20 µM PTER-ITC (for the next 24 h). The cells were then washed with PBS (phosphate-buffered saline) and incubated with 500 µl DAPI (0.5 µg/ml; 10 min, in the dark) and observed by fluorescence microscopy (Zeiss, Axiovert 25).

### Flow cytometry assay for apoptotic cells

PTER-ITC-induced apoptosis in both MCF-7 and MDA-MB-231 cells was determined quantitatively by flow cytometry using the annexin V-conjugated Alexa Fluor 488 Vybrant apoptosis assay kit (V-13241; Molecular Probes, Eugene, OR) following the manufacturer's protocol. Briefly, after cell treatment with 10 and 20 µM PTER-ITC, alone or with PPARγ inhibitor (GW9662) for 24 h, cells were harvested, washed with PBS and incubated with annexin V, Alexa Fluor 488 (Alexa488) and propidium iodide for cell staining in binding buffer (room temperature, 15 min in the dark). Stained cells were analyzed on a fluorescence activated cell sorter (FACS Calibur, BD Biosciences, San Jose, CA) and data were analyzed using Cell Quest 3.3 software.

### Caspase assay

Caspase activity was determined using the ApoTarget caspase colorimetric protease assay sampler kit (KHZ1001; Invitrogen) according to instructions. Briefly MCF-7 and MDA-MB-231 breast cancer cells were treated with 10 and 20 µM PTER-ITC (24 h). Cells were collected, washed in PBS, and lysed in 50 µl lysis buffer (on ice, 10 min). After centrifugation (10,000×g), the supernatant containing 150 µg protein were incubated with 200 µM caspase-3/7 (Ac-DEVD-pNA), caspase-8 (Ac-IETD-pNA) and caspase-9 (Ac-LEHD-pNA) substrates in reaction buffer (37oC, 1 h). Released pNA was measured with a microplate reader (Fluostar Optima) at 405 nm. Increase in caspase-3/7, -8, and -9 activities were determined by direct comparison to level of the uninduced control cells. For caspase inhibitor assays, cells were pretreated with a synthetic pan-caspase inhibitor (20 µM Z-VAD-FMK) and caspase-8 and -9 inhibitors (20 µM Z-IETD-FMK and Z-LEHD-FMK, respectively) for 1 h before addition of 20 µM PTER-ITC for an additional 24 h. This was followed by MTT assay of the samples as above.

### Immunofluorescence staining

For immunofluorescence staining, cells were washed with PBS and fixed in 3% paraformaldehyde, permeabilized with 0.1% Triton X-100 and blocked with 1% BSA (bovine serum albumin; 30 min, room temperature). Cells were then incubated with anti-PPARγ antibody (1∶200 in blocking buffer; 1 h, room temperature). Finally, the cells were washed with PBS and incubated with FITC-labeled anti-rabbit secondary antibody (1∶1000 in blocking buffer; 30 min, room temperature) and observed by fluorescence microscopy (Zeiss, Axiovert 25).

### Luciferase assay

PPARγ activity was studied by luciferase assay as described [18]. Briefly, cells were seeded at density of ∼4×104 cells/well in 12-well microtiter plates, and incubated overnight. Cells were then incubated in serum-free DMEM for ≥1 h before transfection with PPREx3-tk-Luc (three PPRE from rat acyl-CoA oxidase promoter under the control of the Herpes simplex virus thymidine kinase promoter) and Renilla-luc plasmids as an internal control. For PPAR study, cells were transfected with 25 ng pcMX-PPARα, pcMX-PPARβ and pcMX-PPARγ plasmids, each with 250 ng of reporter gene plasmid using Polyfect transfection reagent (Qiagen), according to instructions. Transfected cells were exposed to vehicle, various concentrations of PTER, PTER-ITC and PPAR agonist or antagonist in charcoal-stripped medium (24 h). Cells were then lysed and luciferase activity measured according to kit instructions (Promega, Madison, WI). Triplicates were measured for each experimental point; variability was <10%. Luciferase values for each lysate were normalized to Renilla luciferase activity.

### Oil Red O staining of MCF-7 cells

Approximately 105 cells were cultured on glass coverslips and treated at different PTER-ITC and rosiglitazone concentrations. After 2 days, and every 2 days thereafter, cells were switched to fresh drug-containing medium. MCF-7 cells differentiated for a total of 7 days were washed twice with PBS (pH 7.4) and fixed with 2 ml 10% formalin in PBS (30 min, room temperature). Cells were then washed twice with 2 ml distilled water and stained with 0.5% Oil Red O (Sigma, St. Louis, MO) for 10 min with gentle agitation. Excess stain was removed with 60% isopropanol and cells were washed twice with distilled water before imaging under a light microscope. Accumulated lipids were extracted in 2 ml 100% isopropanol and absorbance measured at 510 nm.

### RT-PCR

Total RNA was extracted from the treated cells using an RNA isolation kit (Genei). Samples were then quantified and equal amounts of the individual treatments were transcribed with the RT-PCR kit (Genei) according to instructions. Similar treatments, followed by RNA isolation and RT-PCR were carried out three times to eliminate inter-assay variations. Primers for PPARγ, PTEN and β-actin were designed using Primer 3 software and standardized in the laboratory. Primer sequences were 5′-TCTGGCCCACCAACTTTGGG-3′ (sense) and 5′-CTTCACAAGCATGAACTCCA-3′ (anti-sense) for PPAR-γ, 5′-ACCAGG ACCAGAGGAAACCT-3′ (sense) and 5′-GCTAGCCTCTGGATTTGACG-3′ (anti-sense) for PTEN and 5′-TCACCCACACTGTGCCCCATCTACGA-3′ (sense) and 5′-CAGCGGA ACCGCTCATTGCCAATGG-3′ (anti-sense) for β-actin. Amplification of PPARγ and PTEN comprised of 29 cycles (PPARγ: 94°C for 60 s, 55°C for 45 s, 72°C for 2 min; PTEN: 94°C for 60 s, 58°C for 45 s, 72°C for 2 min), and for the β-actin control: 25 cycles (94°C for 60 s, 57°C for 45 s, 72°C for 2 min). PCR conditions were optimized to maintain amplification in the linear range to avoid the plateau effect. PCR products were then separated on a 2% agarose gel and visualized in a gel documentation system (BioRad, Hercules, CA). Band intensity on gels was analyzed using ImageJ 1.43 software (NIH, Bethesda, MD) and normalized to β-actin PCR products. Each RT-PCR was carried out three times.

### Western blot analysis

For western blot analysis, lysates were prepared by harvesting cells in lysis buffer [20 mM Tris pH 7.2, 5 mM EGTA, 5 mM EDTA, 0.4% (w/v) SDS and 1X protease inhibitor cocktail]. Protein was quantified with a BCA protein estimation kit (Sigma). Total protein samples (∼40 µg) were analyzed on 12% polyacrylamide gels, followed by immunoblot analysis using a standard protocol. In brief, proteins were transferred to nylon membrane, which was blocked with TBS-T buffer (20 mM Tris-HCl, pH 7.5, 150 mM NaCl, 0.05% Tween-20) containing 5% skim milk powder. The blots were washed with TBS-T buffer and incubated (overnight, 4°C) in the same buffer with primary anti-PPARγ, -PTEN, -survivin, -Bcl-2, -Bax caspase-9 (1∶500) or -β-actin (1∶1000) antibodies (all from Santa Cruz Biotechnology). Blots were then washed and incubated with HRP (horseradish peroxidase)-conjugated anti-rabbit or -mouse secondary antibody (1∶20,000). Color was developed in the dark using the ECL kit (GE Healthcare, Bucks, UK) and blots were analyzed by densitometry with ImageJ 1.43 using β-actin as internal control.

### Molecular docking study

Docking simulations were performed with Glide using the Maestro module of the Schrödinger suite (Suite 2011: Maestro v. 9.2, Schrödinger, New York NY). The crystal structure of PPARγ bound to ligand Telmisartan was used as the starting model (PDB ID 3VN2) [43]. Using the protein preparation wizard, the complex was prepared by addition of hydrogens and sampling at neutral pH. The structure was refined with the optimized potential for liquid simulations (OPLS) 2005 force field [44] and minimized to a root mean square deviation (RMSD) of 0.30 Å. The Telmisartan binding pocket, which lies within the protein ligand-binding domain (LBD; residues 225–505), was identified on the PPARγ/Telmisartan complex and the receptor grid was generated. During this process, no Van der Waal radius sampling was done; the partial charge cut-off was set at 0.25 and no constraints were enforced [45]. Ligands under study were drawn with ChemDraw [46] and 3-D structure files were generated at Online SMILES Translator and Structure File Generator (http://cactus.nci.nih.gov/services/translate/), followed by preparation with the Maestro LigPrep wizard. Each ligand was subjected to a full energy minimization in the gas phase employing OPLS2005 force field [44], with the generation of structures by different combinations of ionized states and considering all possible tautomeric states in a pH range of 5 to 9. Docking calculations were done using the Extra Precision (XP) mode of Glide [47], maintaining the receptor fixed and ligand flexible. This mode incorporates a more refined and advanced scoring function for protein-ligand docking, which gives an overall approximation of the ligand binding free energy. The function is given by
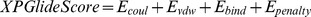
where E_coul_ is coulomb interaction energy; E_vdw_ is Van der Waals interaction energy; E_bind_ is binding energy and E_penalty_ is energy due to disolvation and ligand strain. Finally, post-docking energy minimization was used to improve the geometry of the poses.

### Statistical analysis

Data are expressed as mean ± SEM and statistically evaluated with one-way ANOVA followed by the Bonferroni *post hoc* test using Graph Pad Prism 5.04 software (Graph Pad Software, San Diego CA). A *p* value of <0.05 was considered statistically significant.

## Results

### PPARγ is involved in PTER-ITC-induced inhibition of cell proliferation

MCF-7 and MDA-MB-231 cells were treated with increasing concentrations (1–60 µM) of PTER and PTER-ITC for 24 h and cell survival was determined by MTT assay. Our data showed that treatment of these cells with PTER and PTER-ITC resulted in dose-dependent inhibition of cell proliferation, which was more pronounced after PTER-ITC treatment compared to vehicle-treated control cells (Fig. 1B, C). In MCF-7 cells treated with 10 and 20 µM PTER-ITC, viable cell numbers decreased from 75% to 55%, which was about 92% and 85% respectively, after PTER treatment (Fig. 1D). Preincubation of cells with 10 µM GW9662 (a PPARγ antagonist) increased cell survival from 75% to 87% in the presence of 10 µM PTER-ITC, which was 55% to 67% in the case of 20 µM PTER-ITC (*p*<0.05) (Fig. 1D). PTER treatment did not lead to improvement in viability when cells were pretreated with GW9662. Results were similar for MDA-MB-231 cells, in which with 10 µM GW9662 pretreatment increased cell survival from 82% to 97% in the presence of 10 µM PTER-ITC, and 70% to 87% after 20 µM PTER-ITC treatment (*p*<0.05) (Fig. 1E).

### Differential PPARγ expression in distinct breast cancer cell lines

**Figure 2:**
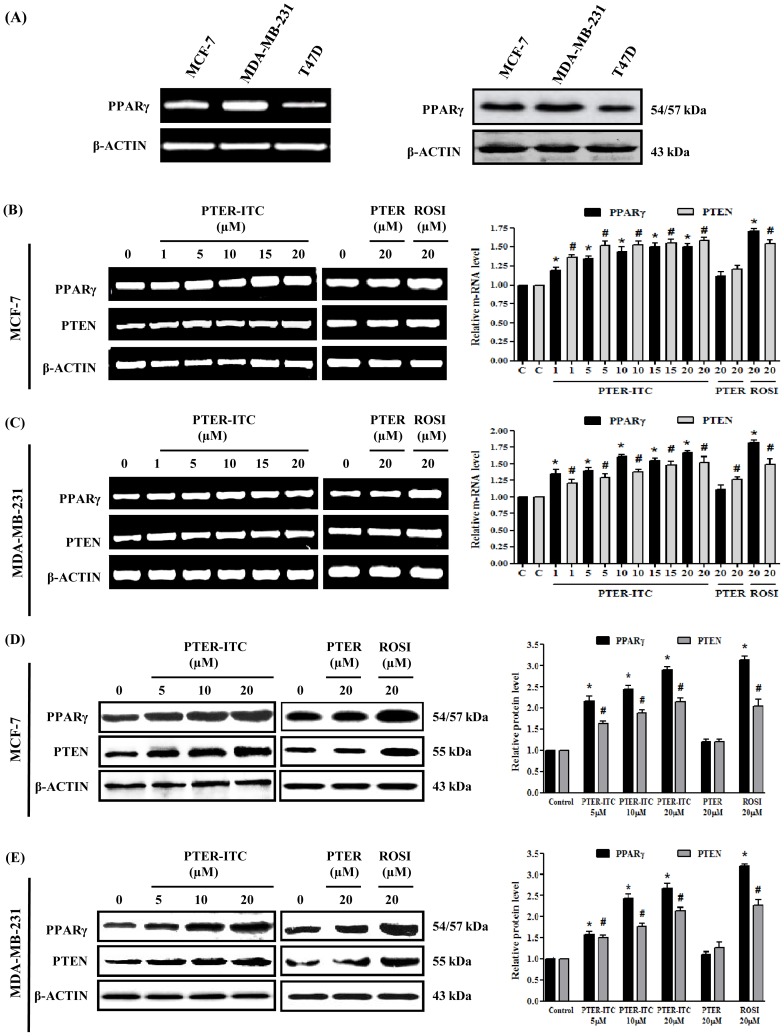
PTER-ITC upregulates PPARγ and PTEN expression levels. (A) PPARγ expression in three breast cancer cell lines as determined by RT-PCR (left) and immunoblot analysis (right). (B) Effect of PTER-ITC, PTER and rosiglitazone on PPARγ and PTEN mRNA expression as determined by RT-PCR in MCF-7 and (C) MDA-MB-231 cells. (D) Effect of PTER-ITC, PTER and rosiglitazone on PPARγ and PTEN protein expression as determined by immunoblot analysis in MCF-7 and (E) MDA-MB-231 cells. Histogram (right panel in each figure) shows relative band intensities normalized to the corresponding β-actin level. Data are expressed as *x*-fold increase relative to control; values shown as mean ± SEM of three independent experiments. * and # indicate statistically significant differences with respect to controls for PPARγ and PTEN proteins, respectively. *p*<0.05; ROSI, rosiglitazone.

Three breast cancer cell lines (MCF-7, MDA-MB-231, T47D) were analyzed for PPARγ expression. RT-PCR results showed that *PPARγ* transcription was highest in MDA-MB-231 cells compared to the other two cell lines (Fig. 2A, left). In accordance, we found that PPARγ protein expression was also higher in MDA-MB-231 cells, followed by MCF-7 and T47D cell lines (Fig. 2A, right). Based on these results, we selected MCF-7 and MD-MB-231 cells as *in vitro* models for the remaining part of the study.

### PTER-ITC upregulates PPARγ expression and activity

**Figure 3:**
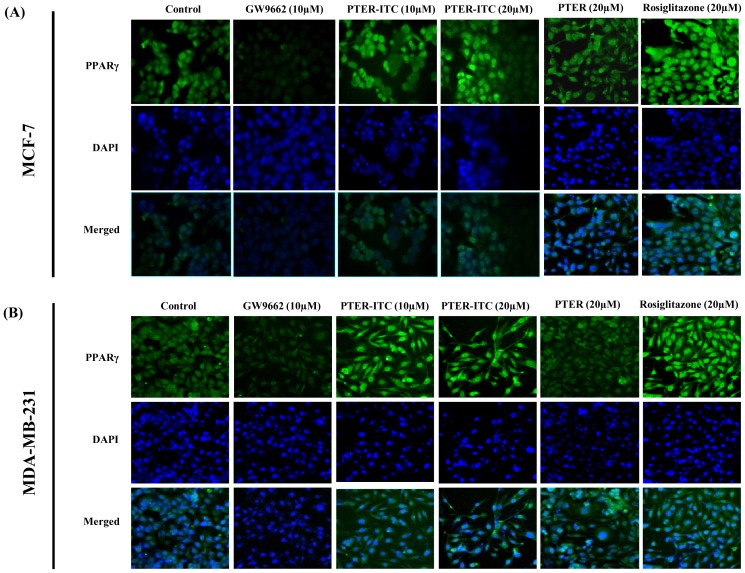
Induction of PPARγ expression in response to different treatments. (A) Immunofluorescence analysis to detect PPARγ protein in MCF-7 and (B) MDA-MB-231 breast cancer cells after treatment with GW9662, PTER-ITC, PTER and rosiglitazone. Figures show one representative experiment of three performed. Magnification, 200×.

To examine changes in PPARγ mRNA and protein expression following exposure to different drugs, we used RT-PCR, immunoblot and immunofluorescence analysis. In MCF-7 cells, the *PPARγ* transcript level increased in response to PTER-ITC in a dose-dependent manner, which was ∼1.5-fold at the highest dose tested (Fig. 2B). In contrast, PTER showed no significant increase, while the PPARγ agonist rosiglitazone caused a 1.7-fold upregulation in its expression, as anticipated. Results were similar in MDA-MB-231 cells, in which PTER-ITC, PTER and rosiglitazone showed 1.6-, 1.1- and 1.8-fold increases in PPARγ mRNA levels at a 20 µM concentration (Fig. 2C). This result was validated by immunoblot analysis, in which we observed a dose-dependent increase in PPARγ protein expression after PTER-ITC treatment in MCF-7 (2.1- to 2.8-fold) and MDA-MB-231 cells (1.5- to 2.6-fold) (Fig. 2D, E) (*p*<0.05). Treatment with 20 µM PTER had little or no effect, while treatment with same dose of rosiglitazone led to a significant increase in PPARγ expression in MCF-7 and MDA-MB-231 cells (p<0.05). Immunofluorescence analysis of PPARγ localization also showed increased nuclear accumulation of PPARγ for PTER-ITC- and rosiglitazone-treated MCF-7 (Fig. 3A) and MDAMB-231 cells (Fig. 3B) compared to control cells, which was markedly inhibited by GW9662. PTER treatment led to no increase in PPARγ expression or activity. These data show that PPARγ expression was upregulated by PTER-ITC at both the transcriptional and translational levels.

### PPARγ participates in PTER-ITC-mediated upregulation of the PTEN tumor suppressor gene

To determine the effect of PTER, PTER-ITC and rosiglitazone on the expression pattern of the tumor suppressor gene PTEN, we treated MCF-7 and MDA-MB-231 cells with various concentrations of drugs for 24 h. RT-PCR and immunoblot analysis showed that PTER-ITC increased PTEN expression at both the transcriptional (Fig. 2B, C) and translational levels (Fig. 2D, E) in a dose-dependent manner (p<0.05). The most effective dose was 20 µM PTER-ITC, which caused an increase almost comparable to that of rosiglitazone. There was little or no difference in the relative level of PTEN in the PTER-treated group compared to controls (Fig. 2D, E) (p<0.05).

### PTER-ITC increased PPARγ and PPARβ activity in MCF-7 cells

**Figure 4:**
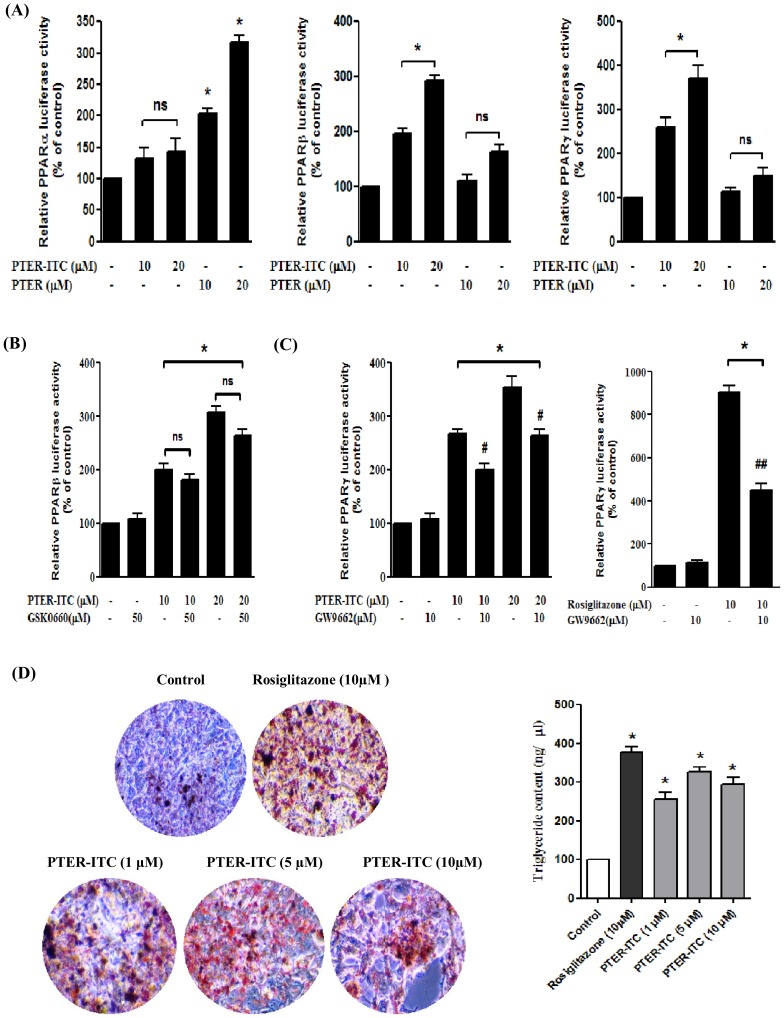
PTER-ITC alters PPAR activity and induces differentiation of MCF-7 cells. (A) Effect of PTER-ITC and PTER on the activity of various PPAR in MCF-7 cells, as determined by transactivation assay. Data are expressed as a percentage of PPAR activity relative to the respective control. Values shown as mean ± SEM of three independent experiments. * indicates significant difference relative to vehicle-treated control; *p*<0.05. (B) Effect of PPARβ inhibitor (GSK0660) and (C) PPARγ inhibitor (GW9662) and activator (rosiglitazone) on PTER-ITC-induced transactivation of PPAR. Cells were transfected with pcMX-PPARβ/γ plasmids, together with PPRE-tk-luc and Renilla plasmids (18 h). Cells were then pre-treated with GSK0660/GW9662 (4 h), followed by PTER-ITC/rosiglitazone treatment (24 h). Data are expressed as percentages of PPARβ/γ activity relative to the vehicle-treated control ( = 100). Values are shown as mean ± SEM of three independent experiments. *, # and ## indicate statistically significant difference compared to respective controls, only PTER-ITC (either 10 or 20 µM) and rosiglitazone-treated groups, respectively; *p*<0.05. ns, not significant. (D) Oil Red O staining showing lipid accumulation in MCF-7 cells treated with different doses of PTER-ITC and rosiglitazone (10 µM), observed by light microscopy (200x). Histogram (right) shows spectrophotometric estimation of intracellular neutral lipids. Values shown as mean ± SEM of two independent experiments. * indicates significant difference relative to vehicle-treated controls; *p*<0.05.

We used a luciferase reporter-based transactivation assay to study the effect of PTER-ITC on the activity of various PPAR types in breast cancer cells. Cells were transfected with plasmids encoding each PPAR protein (pcMX-PPARα, pcMX-PPARβ or pcMX-PPARγ) and with PPRE-tk-Luc and Renilla luciferase plasmids as internal control. Cells were then treated with PTER and PTER-ITC (24 h), followed by extraction of whole-cell lysates for analysis of luciferase activity. PTER-ITC induced PPARβ and PPARγ activities, but had no significant effects on PPARα (Fig. 4A; p<0.05), whereas PTER induced PPARα activity, with no significant change in PPARβ and PPARγ activities (Fig. 4A; p<0.05). We examined the specificity of PTER-ITC on PPARγ and PPARβ activity, using their respective agonists and antagonists. The PPARβ antagonist GSK0660 did not reverse PTER-ITC-induced PPARβ activity (Fig. 4B), suggesting that the PTER-ITC effect on PPARβ was non-specific. The PPARγ antagonist GW9662 reversed PTER-ITC-induced PPARγ activity significantly (Fig. 4C, left), as well as the activity of rosiglitazone, a PPARγ agonist (Fig. 4C, right). These data suggest that PTER-ITC activity is mediated via the PPARγ but not the PPARβ pathway.

### Effects of PTER-ITC on MCF-7 cell differentiation

PPARγ activation induces cells to a more differentiated, less malignant state and causes extensive lipid accumulation in cultured breast cancer cells [30]. We thus used Oil Red O staining to test whether addition of PTER-ITC and rosiglitazone in MCF-7 cells also induces differentiation. Untreated MCF-7 cells showed nominal lipid accumulation as measured by Oil Red O staining (Fig. 4D, left). In contrast, rosiglitazone treatment (10 µM) strongly induced lipid accumulation; PTER-ITC treatment also caused a dose-dependent increase in lipid accumulation, albeit to a lesser extent than rosiglitazone (Fig. 4D). Maximum lipid accumulation was found at 5 µM PTER-ITC (Fig. 4D, right).

### Molecular modeling of PPARγ LBD/PTER-ITC binding

**Figure 5:**
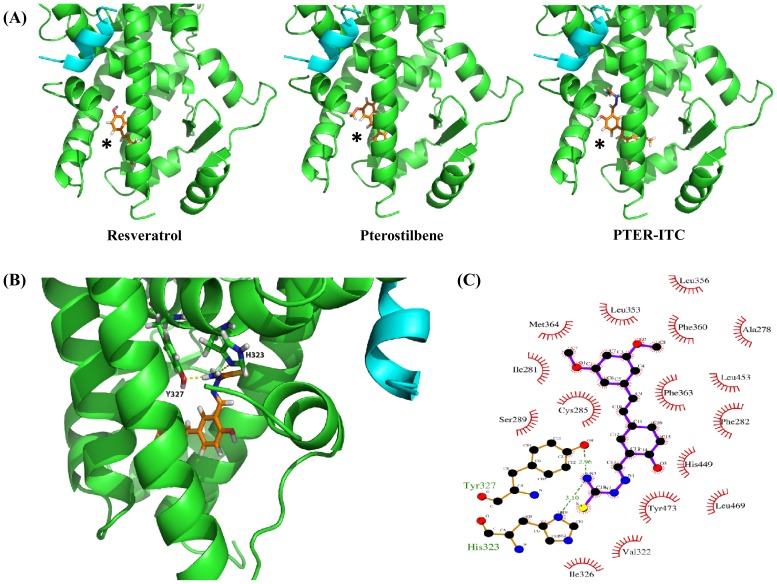
Analysis of PTER-ITC docking pattern with PPARγ. (A) Mode of binding of resveratrol, PTER and PTER-ITC to PPARγ. Note the distinct orientations of the ligands. The broad range of ligand binding ability of PPARγ can be explained in part by the large T-shaped ligand binding area, which permits ligands to adopt distinct orientations (figures generated with PyMOL molecular graphics system). (B) Interaction of PTER-ITC within the ligand-binding pocket. Residues H323 and Y327 of protein chain A are involved in hydrogen bond formation with N3 of the ligand. Yellow dashed lines indicate bonding; interacting residues are labeled. (C) Ligand interaction plot showing different hydrophobic and two hydrogen bond interactions of PTER-ITC with PPARγ. Hydrogen bonds are indicated by green dashed lines, with their respective distances.

Since PTER-ITC increased PPARγ transactivation by acting as a selective PPARγ ligand, we used molecular docking analysis to further study PPARγ LBD (ligand-binding domain)/PTER-ITC interaction at the cellular level. PTER-ITC, its parent compound (PTER), and resveratrol were docked into the PPARγ LBD (see Methods); the binding mode of each ligand to PPARγ LBD is shown in Fig. 5A, with their respective docking scores and interaction energies in Table 1. The terms “XP Glidescore or docking score” and “Emodel” were used to denote interactions between ligand and receptor. Based on these two scores, we observed that the PTER-ITC molecule might have better binding affinity for PPARγ (Table 1). In terms of interaction with different residues, PTER-ITC showed better performance than PTER and resveratrol. In the best-docked position, PTER-ITC formed two hydrogen bonds with the receptor, involving residues His323 and Tyr327 (Table 1; Fig. 5B). In addition, through extensive hydrophobic interactions, it bound more firmly to the receptor than the other two ligands (Fig. 5C). Tyr473 is involved in hydrogen bond formation with both PTER and resveratrol, indicating a similar orientation of the two molecules, which is also evident from close analysis of their docking positions (Fig. 5A). Besides hydrogen bonds and hydrophobic interactions, PTER-ITC is also involved in the formation of π-π stacking between LBD residues His449 and Phe282 and their central benzene rings. This stacking could stabilize PTER-ITC after binding and strengthen the interaction. Similar stacking is partially observed in PTER, which involves only His449.

**Table 1:** Hydrogen bonds and hydrophobic interactions between ligand and PPAR-γ ligand binding domain (LBD).

Ligand	Hydrogen bonds^a^	Hydrophobic contacts^b^	Evdw^c^	Ecoul^d^	Emodel^e^	Docking score
**PTER-ITC**	HIS323 (3.10), TYR327(2.96)	ILE281 PHE282, CYS285, ILE326, LEU353, LEU356, PHE360, PHE363, MET364, HIS449, TYR473	−43.4	−6.7	−53.4	−8.46
**PTER**	TYR473 (2.87)	CYS285, SER289, PHE360, PHE363, HIS449, TYR479	−21.4	−1.8	−30.5	−6.78
**Resveratrol**	TYR473 (3.05)	PHE282, CYS285, SER289, PHE360, PHE363, TYR473	−23.2	−3.8	−34.3	−7.30

Average Van der Waals (Vdw), Electrostatic (Coul) and model energy (Emodel) of ligands after docking. The corresponding docking scores are also mentioned.

a Bond length in Å is given under parentheses. ^b^Only strong hydrophobic contacts forming residues are depicted. ^c^Evdw =  Van Der Waals interaction energy. ^d^Ecoul  =  Coulomb interaction energy. ^e^Emodel  =  Model energy.

### PPARγ antagonist GW9662 inhibits PTER-ITC-induced apoptosis

**Figure 6:**
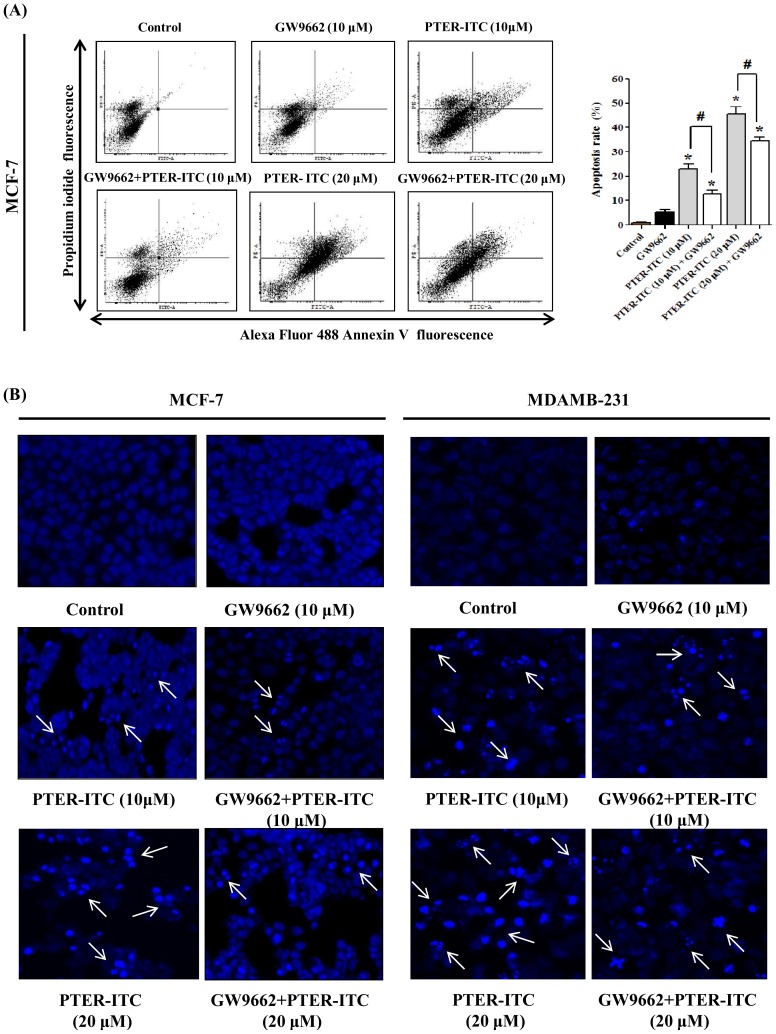
PTER-ITC induces PPARγ-dependent apoptosis in breast cancer cells. (A) Representative FACS analysis of cells using annexin V as marker. Histogram (right) shows the apoptosis rate induced by PTER-ITC alone and in the presence of GW9662. Values are mean ± SEM from three independent experiments. * and # indicate statistically significant differences compared to vehicle-treated control and only PTER-ITC treated groups, respectively; *p*<0.05. (B) Apoptosis induced by PTER-ITC alone and in the presence of GW9662, visualized by fluorescence microscopy using DNA-binding fluorochrome DAPI in MCF-7 and MDA-MB-231 breast cancer cells. Figures show a representative experiment of three performed. Magnification, 200×. Arrows indicate the formation of apoptotic bodies.

We analyzed PTER-ITC apoptosis induction by flow cytometry, using annexin V and propidium iodide (PI) double staining to assess the cause of decreased cell survival after PTER-ITC treatment. We incubated MCF-7 cells with varying concentrations of PTER-ITC, alone or with GW9662 (10 µM; 24 h). PTER-ITC treatment significantly increased the percentage of apoptotic cells, and the effect was partly attenuated by pre-incubation with GW9662 (Fig. 6A; *p*<0.05). Results were similar for MDA-MB-231 cells (not shown). PTER-ITC also induced apoptosis-associated morphological changes, as cells with condensed nuclei and nuclear fragmentation were apparent after treatment (Fig. 6B), which was minimal in vehicle-treated MCF-7 and MDA-MB-231 cells. The apoptotic nuclear changes were clearly reduced in cells pre-treated with 10 µM GW9662 (Fig. 6B). These data suggest that blockade of PPARγ activity blunted the drug-induced cell apoptosis.

### PTER-ITC induces caspase-dependent apoptosis

**Figure 7:**
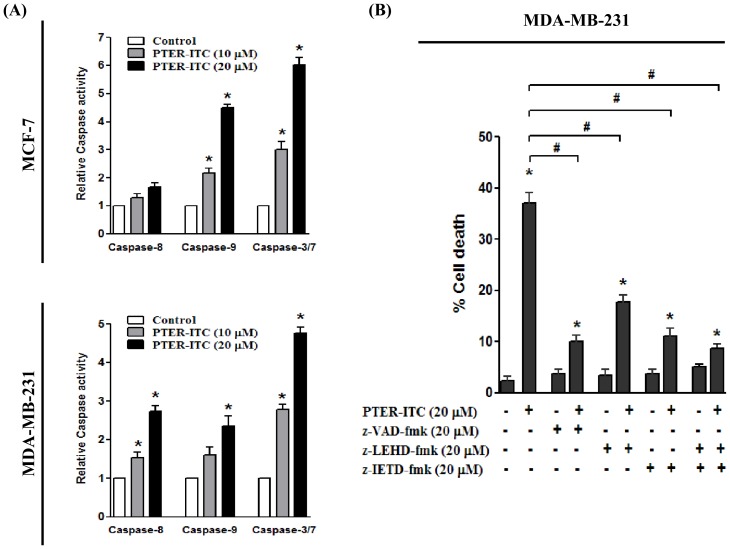
PTER-ITC induces caspase-dependent apoptosis in breast cancer cells. (A) Effects of 10 and 20 µM PTER-ITC on caspase-8, -9 and -3/7 activities in MCF-7 and MDA-MB-231 cells. Results are the mean ± SEM of three independent experiments. * indicates statistically significant difference relative to respective controls; *p*<0.05. (B) Effect of caspase inhibitors on PTER-ITC-induced apoptosis in MDA-MB-231 cells. Data shown as mean ± SEM of three independent experiments. * and # indicate statistically significant difference with respect to control and only PTER-ITC-treated cells, respectively; *p*<0.05.

Apoptosis is a complex activity that mobilizes a number of molecules, and its mechanisms are classified as caspase-dependent or -independent. The caspase-dependent pathway can be further divided into extrinsic or intrinsic pathways, determined by involvement of caspase-8 or caspase-9, respectively. Both of these pathways involve activation of caspase-3/7, which is important for inducing downstream molecules responsible for DNA cleavage. To further examine the mechanism that underlies PTER-ITC-induced death of breast cancer cells, we studied a possible role for caspase in this process by measuring the enzymatic activity of caspase-3/7, -8 and -9. We observed a gradual increase in caspase-9 and caspase-3/7 activities in MCF-7 and MDA-MB-231 cells treated with 10 and 20 µM PTER-ITC for 24 h (Fig. 7A). In contrast, there were no significant changes in caspase-8 activity in MCF-7 cells, whereas we found a dose-dependent increase in activity in MDA-MB-231 cells. Our data thus suggest that PTER-ITC induced activation of the intrinsic caspase pathway in MCF-7 cells, while it induced both extrinsic and intrinsic caspase pathways in MDA-MB-231 cells.

To determine whether caspase activation was involved in PTER-ITC-induced death of cultured breast cancer cells, we used pharmacological caspase inhibitors to test whether they protect cells from undergoing apoptosis. In the case of MDA-MB-231 cells, the general caspase inhibitor Z-VAD-FMK inhibited apoptosis most efficiently (up to 70–80%; Fig. 7B, p<0.05), suggesting that apoptosis is the predominant form of cell death induced by PTER-ITC in these cells. Z-LEHD-FMK, a specific inhibitor of caspase-9, inhibited PTER-ITC-induced apoptosis by 50–55% (p<0.05), while Z-IETD-FMK, a specific inhibitor of caspase-8, inhibited PTER-ITC-induced apoptosis by 65-70% (p<0.05). In contrast, Z-LEHD-FMK inhibited PTER-ITC-induced apoptosis by 66–70% in MCF-7 cells, while Z-IETD-FMK did not effectively block PTER-ITC-induced apoptosis in this cell line, which confirmed previous reports [41]. Our data thus demonstrate that PTER-ITC-induced apoptosis is a caspase-dependent process that involves both caspase-8 and -9 in MDA-MB-231 cells and only caspase-9 in MCF-7 cells.

### MAPK and JNK are involved in PTER-ITC-induced PPARγ activation and apoptosis

**Figure 8:**
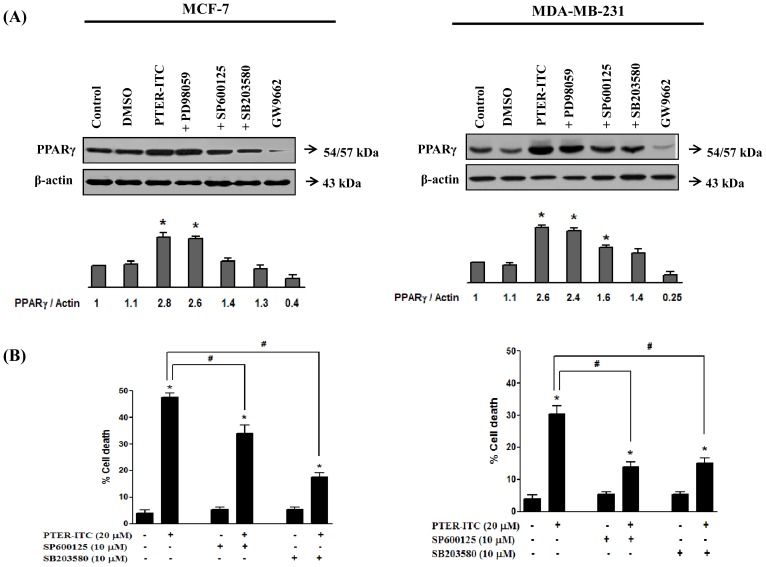
PTER-ITC alters PPARγ activity through p38 MAPK and JNK pathways. (A) PTER-ITC induces PPARγ expression through p38 MAPK and JNK pathways in MCF-7 and MDA-MB-231 cells. The experiment was performed in duplicate and yielded similar results. Histogram (bottom) shows relative band intensities normalized to the corresponding β-actin level, where the vehicle-treated group = 1. (B) Effects of p38 MAPK and JNK inhibitors on PTER-ITC-induced apoptosis in MCF-7 and MDA-MB-231 cells. Data are shown as mean ± SEM of three independent experiments. * and # indicate statistically significant difference with respect to vehicle-treated control and only PTER-ITC treated cells, respectively; *p*<0.05.

To test for a role of MAPK (mitogen-activated protein kinase) in PTER-ITC-induced PPARγ activation and apoptosis of breast cancer cells, we pre-treated MCF-7 and MDA-MB-231 cells with 20 µM ERK inhibitor (PD98059), 10 µM JNK inhibitor (SP600125) or 10 µM p38 MAPK inhibitor (SB203580) for 1 h, followed by PTER-ITC treatment for an additional 24 h. Total proteins were then isolated for analysis of PPARγ expression patterns. In both breast cancer cell lines, SB203580 and SP600125 pre-treatment completely blocked PTER-ITC-induced PPARγ expression, whereas pre-treatment with PD98059 or DMSO had no effect (Fig. 8A). We therefore suggest that PTER-ITC induces p38 MAPK and JNK pathways to upregulate PPARγ expression in MCF-7 and MDA-MB-231 cells.

Since both p38 MAPK and JNK pathways had important roles in PTER-ITC-induced PPARγ expression, we evaluated whether inhibition of either pathway protected cells from PTER-ITC-induced apoptosis. The breast cancer cells were pre-treated with 10 µM SB203580 (p38 MAPK inhibitor) or SP600125 (JNK inhibitor) for 1 h, followed by PTER-ITC treatment for an additional 24 h, and the percentage of dead cells was determined in an MTT assay. In the case of MCF-7 cells, SB203580 pre-treatment abolished PTER-ITC-induced cell death, which was only partially blocked by the JNK inhibitor (SP600125) (Fig. 8B). For MDA-MB-231 cells, inhibition of both p38 MAPK and JNK pathways abolished PTER-ITC-induced cell death. These results confirmed involvement of both p38 MAPK and JNK pathways in PTER-ITC-induced PPARγ activation and apoptosis in MCF-7 and MDA-MB-231 cells, albeit to a lesser extent by the JNK pathway in MCF-7 cells.

### PTER-ITC induces apoptosis by targeting PPARγ-related proteins

**Figure 9:**
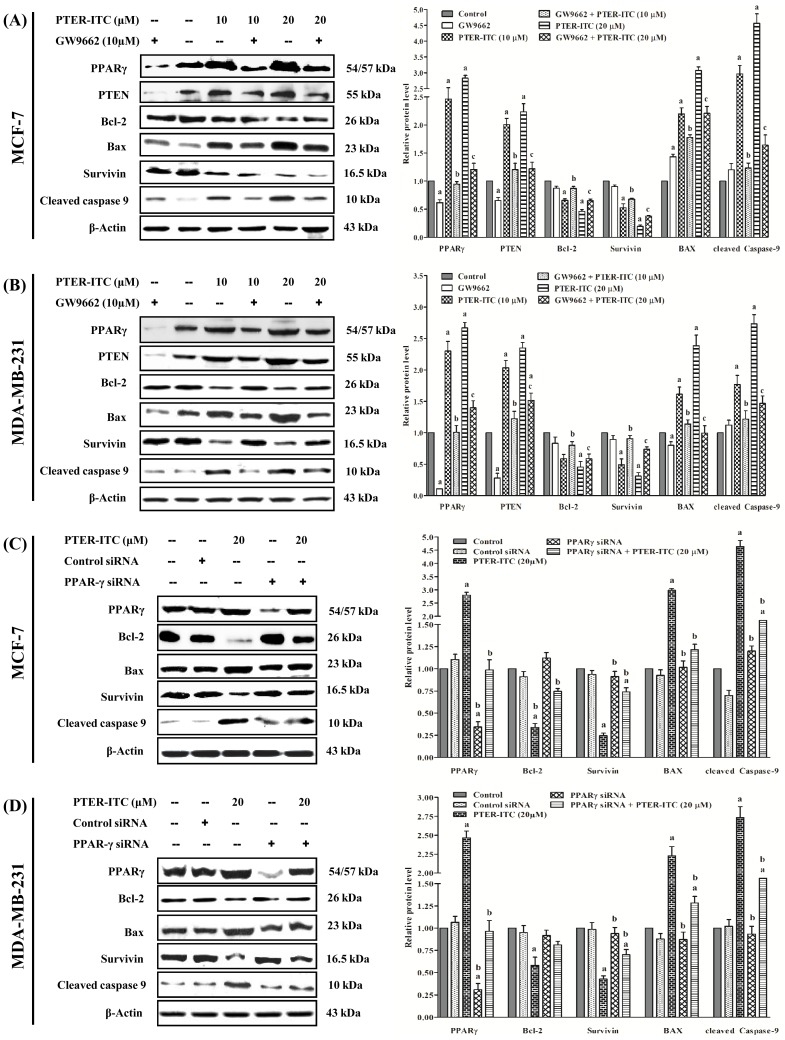
PTER-ITC induces apoptosis by targeting PPARγ-related proteins. Immunoblot analysis for apoptotic markers and PPARγ-regulated genes in response to PTER-ITC and GW9662 treatment in (A) MCF-7 and (B) MDA-MB-231 cells. Cells were pre-treated with 10 µM GW9662 (1 h) before treatment with 10 and 20 µM PTER-ITC (24 h). Whole-cell extracts were resolved by SDS-PAGE and probed with indicated antibodies. Expression levels of samples were normalized to the corresponding β-actin levels. Histogram (right panels in each figure) show data expressed as *x*-fold change relative to control; bars show mean ± SEM of three independent experiments. a, b and c indicate significant levels of differences with respect to control, 10 and 20 µM only PTER-ITC-treated groups, respectively, for each protein. *p*<0.05. (C) Effect of PPARγ siRNA on PTER-ITC-induced apoptosis of MCF-7 and (D) MDA-MB-231 cells. Both cells were transfected with PPARγ siRNA (final concentration 100 nM). After 24 h, cells were treated with 20 µM PTER-ITC and incubated (24 h). Levels of PPARγ-related proteins were detected in cell lysates by immunoblot analysis. Histogram (right panel in each figure) shows relative band intensities normalized to the corresponding β-actin level. Data are expressed as *x*-fold change relative to control; bars show mean ± SEM of three independent experiments. a and b indicate significant differences with respect to vehicle-treated control and only 20 µM PTER-ITC-treated groups, respectively; *p*<0.05.

To elucidate the mode of action of PTER-ITC as an apoptotic agent in the PPARγ-dependent pathway, we studied its effect on the regulation of PPARγ-related genes in both breast cancer cell lines. PTER-ITC significantly increased PPARγ, PTEN and Bax, and decreased Bcl-2 expression in a dose-dependent manner both at the level of transcription (not shown) and translation (Fig. 9A, B). Moreover, PTER-ITC significantly decreased expression of survivin, which blocks caspase-9 and -3, thereby inhibiting apoptosis.

To determine whether the increase in apoptosis and decrease in PPARγ-related genes was due to PTER-ITC-induced PPARγ activation, we performed two sets of experiments. First, we used the PPARγ antagonist GW9662 to block PPARγ pathway activation, followed by 24 h PTER-ITC treatment. Second, PPARγ protein expression was knocked down in MCF-7 and MDA-MB-231 cells by transfection of PPARγ siRNA, followed by 24 h PTER-ITC treatment. Our results showed that MCF-7 and MDA-MB-231 cells in both treatment protocols restored the inhibition of Bcl-2 and survivin caused by PTER-ITC alone (Fig. 9A–D). In addition, PTER-ITC upregulated Bax and PTEN protein expression in a dose-dependent manner, which was inhibited by the PPARγ antagonist or PPARγ siRNA (Fig. 9A–D), indicating that PTER-ITC modulation of Bax and PTEN is PPARγ-dependent. Furthermore, PTER-ITC induction of cleaved caspase-9 in both MCF-7 and MDAMB-231 cells was attenuated by GW9662 or PPARγ siRNA treatment (Fig. 9). These data suggest that PTER-ITC induced PPARγ expression, which subsequently enhanced expression of downstream components of this pathway, finally leading to apoptosis.

## Discussion

Breast cancer is the most commonly diagnosed cancer and the second leading cause of cancer death [48]. The mortality rate of breast cancer is high because of disease recurrence, which remains the major therapeutic barrier in this cancer type. Although many cytotoxic drugs have been developed for clinical use, cancer chemotherapy is always accompanied by adverse effects, which can be fatal in some cases. Due to the lack of satisfactory treatment options for breast cancer to date, there is an urgent need to develop preventive approaches for this malignancy. There is a growing interest in combination therapy using multiple anticancer drugs that affect several targets/pathways. A single molecule containing more than one pharmacophore, each with a different mode of action, could be beneficial for cancer treatment. Here, we studied the effectiveness of a new synthetic derivative of pterostilbene, a phytochemical isolated from *Pterocarpus marsupium* stem heart wood, in hormone-dependent (MCF-7) and -independent (MDA-MB-231) breast cancer cell lines.

PPARγ is widely expressed in many tumors and cell lines, and has become a promising target for anticancer therapy. This nuclear receptor has a critical role in breast cancer proliferation, survival, invasion, and metastasis [13], [18], [20], [21], [25]–[28]. The effectiveness of PPARγ agonists as anticancer agents has been examined in various cancers including colon, breast, lung, ovary and prostate [49]. We tested whether PTER-ITC mediates its anti-proliferative and pro-apoptotic effects in breast cancer cells through activation of the PPARγ signaling cascade. Our results showed that PTER-ITC activated PPARγ expression in a dose-dependent manner, followed by downregulation of its anti-apoptotic genes (Bcl-2 and survivin) to induce noteworthy levels of apoptosis in hormone-dependent (MCF-7) and -independent (MDA-MB-231) breast cancer cells.

The PTER-ITC conjugate can be considered more advantageous than existing PPARγ ligands such as rosiglitazone or pioglitazone for breast cancer treatment, as PTER-ITC causes more pronounced cell death at a much lower dose than other ligands [50]–[52]. In addition, most (if not all) the other ligands are estrogenic in nature [53], and could thus act as positive factors for ER-dependent breast, ovary and uterine cancers, whereas PTER-ITC is anti-estrogenic at the dose used for this study. Considering these two major points, we consider that the drug could be used at much lower concentrations, which might help reduce the side effects reported for most other PPARγ ligands. PTER-ITC molecule nonetheless requires further validation before use in clinical trials that target the PPARγ pathway.

The most important characteristic of a cancer cell is its ability to sustain proliferation [54]. The pathways that control proliferation in normal cells are altered in most cancers [55]. We thus analyzed the PTER-ITC effect on proliferation of breast cancer cells, and found that PTER-ITC caused significant, dose-dependent inhibition of breast cancer cell growth *in vitro*. This effect was partially reversed, however, when PTER-ITC was combined with PPARγ antagonists. This result suggests that the PTER-ITC anticancer effects are mediated through the PPARγ activation pathway. These data coincide with findings in several *in vivo* and *in vitro* studies in which PPARγ agonists such as rosiglitazone or troglitazone decreased proliferation of breast cancer cell lines, mediated in part by a PPARγ-dependent mechanism [26], [56].

To elucidate the molecular mechanisms that underlie the anticancer effects observed for PTER-ITC, we studied its effect on activation of PPARγ. To the best of our knowledge, this is the first report showing PTER-ITC participation in the PPARγ-dependent signaling pathway. Our data show that PTER-ITC increased PPARγ transcriptional and translational activity in MCF-7 and MDA-MB-231 cells. To establish the essential role of PTER-ITC in PPARγ-mediated apoptosis of breast cancer cells, we used PPARγ siRNA and its drug antagonist to inhibit PPARγ signaling, and demonstrated apoptosis prevention and caspase activation. We also observed an increase in PPARβ activity after PTER-ITC treatment, with no significant reduction after antagonist treatment, suggesting that the increase was non-specific. Although some earlier studies reported involvement of PPARβ activity in tumorigenesis, many others contradicted this idea. The PPARβ ligand GW501516 was reported to promote human hepatocellular growth [57], although another study showed that certain PPARβ ligands such as GW0742 and GW501516 reduced growth of MCF-7 and UACC903 cell lines [58]. The role of PPARβ in cancer therapeutics is therefore complex and not yet fully defined [59]. Hence the relationship between PTER-ITC and PPARβ could provide an alternative platform to study the involvement of this pathway in cancer therapy.

PPARγ is a phosphoprotein, and many kinase pathways, such as cAMP-dependent protein kinase (PKA), AMP-activated protein kinase (AMPK) and mitogen-activated protein kinase (MAPK) such as ERK, p38 and JNK, have been implicated in the regulation of its phosphorylation [60], [61]. Phosphorylation notably inhibits PPARγ ligand-independent and -dependent transcriptional activation [60], [61]. Research showed that PPARγ agonists activate different MAPK subfamilies, depending on cell type [62]–[65] and that these kinases are involved in cell death [66]–[69]. The role of MAPK signaling pathways in cell death induced by PPARγ agonists is controversial. According to certain studies, PPARγ agonist-induced ERK activation mediates anti-apoptotic signaling [64], while others showed its involvement in inducing cell death [66], [70]. p38 activation by PPARγ agonists is also reported to be regulated differently in various cell types. PPARγ agonists induce p38 activation, leading to apoptosis of cancer cells have been reported in chondrocytes [64], human lung cells [68], liver epithelial cells [62] and skeletal muscle [71]. This coincides with our data, where using pharmaceutical inhibitors, we show that activation of p38 and JNK pathways, but not of ERK, is necessary and sufficient to phosphorylate PPARγ and cause subsequent apoptosis in the breast cancer cell lines studied. At present, we do not know whether PTER-ITC activates p38 and JNK directly, or if it activates other cellular kinase pathways such as PKA and AMPK, which in turn could activate MAPK. Further validation is needed to conclusively establish the pathway(s) involved.

PTEN is a tumor suppressor gene involved in the regulation of cell survival signaling through the phosphatidylinositol 3-kinase (PI3K)/Akt pathway [72]. PI3K/Akt signaling is required for an extremely diverse array of cellular activities that participate mainly in growth, proliferation, apoptosis and survival mechanisms [73], [74]. Activated Akt protects cells from apoptotic death by inactivating compounds of the cell death machinery such as procaspases [73]. PTEN exercises its role as a tumor suppressor by antagonizing the PI3K/Akt pathway [73]. The PPARγ-dependent increase in PTEN caused by PTER-ITC in our experiments not only indicates that the tumor suppressor gene contributes to the growth-inhibitory activities of the compound, but might also trigger its pro-apoptotic actions.

Our results further showed that PTER-ITC downregulated PPARγ-related genes, including Bcl-2 and survivin. These genes are commonly associated with increased resistance to apoptosis in human cancer cells [75]. PTER-ITC-induced PPARγ activation was reduced in the presence of GW9662, together with reversal of decreased survivin and Bcl-2 levels. Furthermore, molecular docking analysis suggested that PTER-ITC could interact with amino acid residues within the PPARγ-binding domain, including five polar and eight non-polar residues within the PPARγ ligand-binding pocket that are reported to be critical for its activity. Together these results suggest that PTER-ITC can be considered a PPARγ agonist, and the survivin and Bcl-2 decrease is due to activation of the PPARγ pathway by PTER-ITC.

Two cellular pathways, differentiation and apoptosis, are the main focus in the development of anti-cancer therapies. Induction of differentiation is one potent mechanism by which some cancer therapeutic and chemopreventive agents act [76]–[78]. Lipid accumulation in MCF-7 cells is supported by the fact that tamoxifen and a few other anti-cancer agents such as ansamycins and suberoylanilide hydroxamic acid induce high lipid production (as high as 5-fold in the case of ansamycins) and by triglyceride accumulation, which results in MCF-7 cell differentiation to a more epithelial-like morphology [79]–[81]. In a previous study, we showed that long-term exposure to PTER causes growth arrest in MCF-7 cells, which might be linked to mammary carcinoma cell differentiation into normal epithelial cell-like morphology and activation of autophagy [38]. In the present study, PTER-ITC also caused differentiation of MCF-7 cells, albeit to a higher level compared to its parent compound PTER than previously reported [38]. Based on these data, it can thus be suggested that PTER-ITC inhibits MCF-7 cell growth mainly through apoptosis, while it can also induce differentiation of these breast cancer cells.

## Conclusions

**Figure 10:**
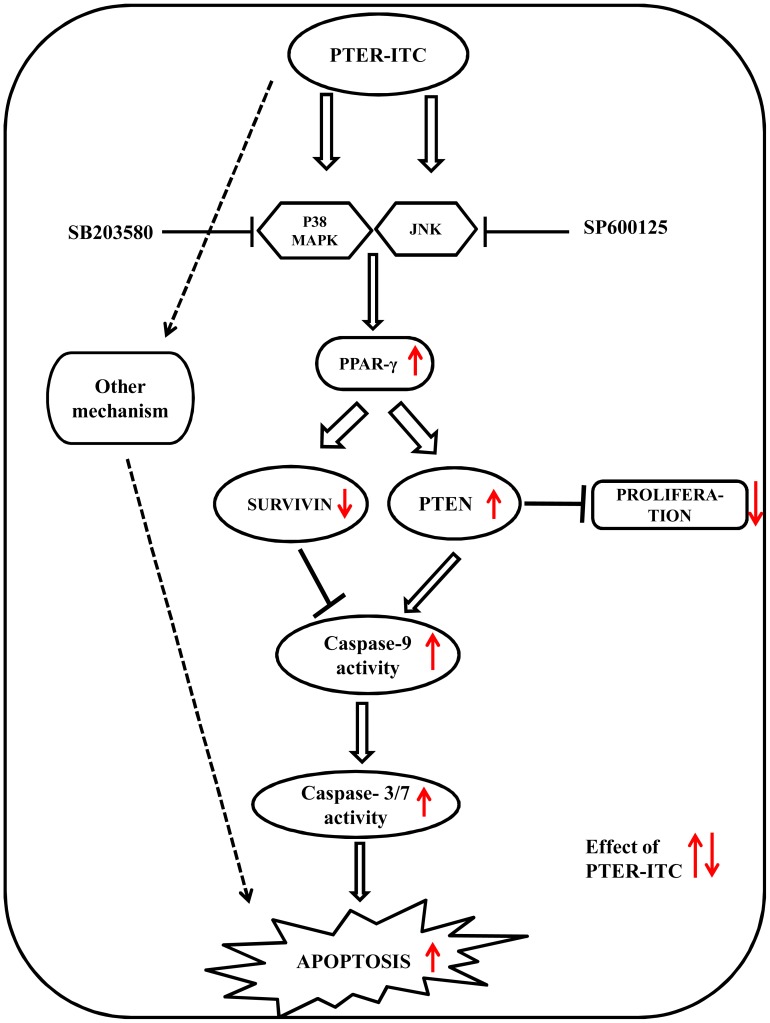
Possible mode of action of PTER-ITC-induced apoptosis and cell growth inhibition in MCF-7 cells. PTER-ITC activates p38 MAPK and JNK, which in turn up regulate PPARγ expression and receptor activity. PPARγ decreases survivin expression and up regulates PTEN expression, both of which increase caspase-9 activity, leading to increased caspase-3/7 activity, which finally results in cell death.

In conclusion, this study highlights the anticancer effects of the novel conjugate of PTER and ITC, and shows that the mechanism involves activation of the PPARγ pathway via PTER-ITC binding to the receptor, which affects its regulated gene products (Fig. 10). PTER-ITC induces apoptosis by enhancing expression of PPARγ genes at both transcriptional and translational levels, which appears to be triggered at least in part by modulation of PTEN. In addition, activation of caspase-9 and downregulation of Bcl-2 and survivin contribute to PTER-ITC-induced cell death. PTER-ITC exhibits differentiation-promoting as well as anti-proliferative effects on MCF-7 cells. Together these results suggest that the PTER-ITC conjugate acts as a PPARγ agonist and is a promising candidate for cancer therapy, alone or in combination with existing therapies. These preliminary data show that further studies are warranted in *in vitro* and *in vivo* models to elucidate the exact mode of action responsible for the effects of this compound.
